# Surpassing the no-cloning limit with a heralded hybrid linear amplifier for coherent states

**DOI:** 10.1038/ncomms13222

**Published:** 2016-10-26

**Authors:** Jing Yan Haw, Jie Zhao, Josephine Dias, Syed M. Assad, Mark Bradshaw, Rémi Blandino, Thomas Symul, Timothy C. Ralph, Ping Koy Lam

**Affiliations:** 1 Centre for Quantum Computation and Communication Technology, Department of Quantum Science, Research School of Physics and Engineering, The Australian National University, Canberra, ACT 2601, Australia; 2 Centre for Quantum Computation and Communication Technology, School of Mathematics and Physics, University of Queensland, St. Lucia, Queensland 4072, Australia

## Abstract

The no-cloning theorem states that an unknown quantum state cannot be cloned exactly and deterministically due to the linearity of quantum mechanics. Associated with this theorem is the quantitative no-cloning limit that sets an upper bound to the quality of the generated clones. However, this limit can be circumvented by abandoning determinism and using probabilistic methods. Here, we report an experimental demonstration of probabilistic cloning of arbitrary coherent states that clearly surpasses the no-cloning limit. Our scheme is based on a hybrid linear amplifier that combines an ideal deterministic linear amplifier with a heralded measurement-based noiseless amplifier. We demonstrate the production of up to five clones with the fidelity of each clone clearly exceeding the corresponding no-cloning limit. Moreover, since successful cloning events are heralded, our scheme has the potential to be adopted in quantum repeater, teleportation and computing applications.

## Abstract (web summary)

Cloning an unknown quantum state is challenging and the limit on the quality of clones is set by the no-cloning theorem. Here, the authors demonstrated the surpassing of such a limit using an effective quantum cloner based on a hybrid probabilistic-deterministic linear amplifier.

## Graphical abstract

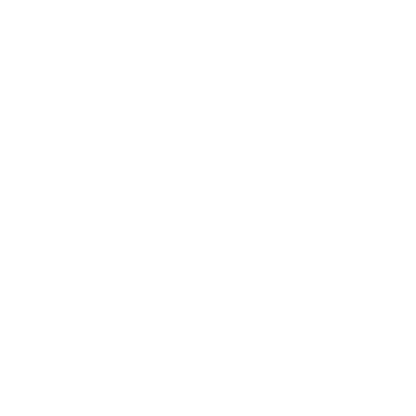

The impossibility to perfectly duplicate an unknown quantum state deterministically, known as the no-cloning theorem^1^, lies at the heart of quantum information theory and guarantees the security of quantum cryptography^2^,^3^. This no-go theorem, however, does not rule out the possibility of imperfect cloning. The idea of generating approximate copies of an arbitrary quantum state was conceived by Buzek and Hillery in their seminal work^4^ with the proposal of universal quantum cloning machine. This discovery has since sparked intense research in both discrete^5^,^6^,^7^,^8^ and continuous variable^9^,^10^,^11^,^12^,^13^ systems to explore the fundamental limit of cloning fidelity allowed by quantum mechanics, known as the no-cloning limit. Several quantum cloning experiments approaching the optimal fidelity enforced by this limit have since been demonstrated for single photons^14^, polarization states^15^ and coherent states^16^.

By forgoing determinism, perfect cloning is not entirely forbidden by the law of quantum physics. In fact, if the quantum states to be cloned are chosen from a discrete, linearly independent set, then the unitarity of quantum evolution does allow probabilistic exact cloning^17^,^18^,^19^,^20^,^21^. Non-deterministic high-fidelity cloning of linearly dependent input states can also be performed if the cloning operation is only arbitrarily close to the ideal case^20^,^22^. Recently, the invention of probabilistic noiseless linear amplifier (NLA)^23^, and its subsequent theoretical studies^24^,^25^,^26^,^27^,^28^ and implementations^29^,^30^,^31^,^32^,^33^ in principle provided a method for cloning arbitrary distributions of coherent states with high-fidelity via an amplify-and-split approach^29^. In practice, however, implementing NLA for coherent states with amplitude |*α*|≥1 remains a technical challenge. This is because the resources required scales exponentially with the coherent state size.

In this article, we follow a different path by adopting a method that interpolates between exact-probabilistic and approximate-deterministic cloning^34^. We show that a hybrid linear amplifier, comprising of a probabilistic NLA and an optimal deterministic linear amplifier (DLA)^16^,^35^, followed by an *N*-port beam splitter is an effective quantum cloner. Previously, Müller *et al*.^36^ demonstrated probabilistic cloning of coherent states which outperformed the best deterministic scheme for input alphabet with random phases but fixed mean photon number. Here, we propose a high-fidelity heralded cloning for arbitrary distributions of coherent states and experimentally demonstrate the production of *N* clones with fidelity that surpasses the Gaussian no-cloning limit *F*_*N*_=*N*/(2*N*−1)^12^,^13^.

## Results

### Hybrid cloning machine

**Figure 1:**
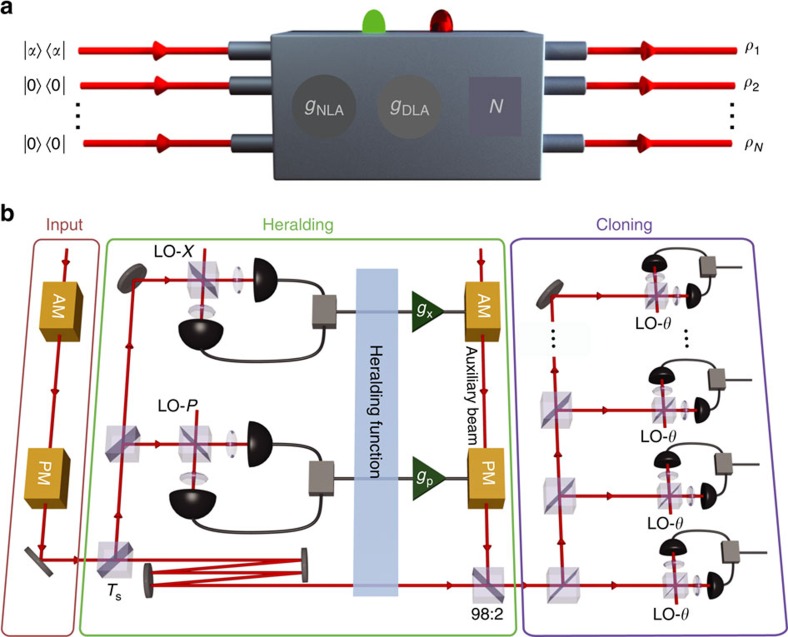
Hybrid Cloning Machine. (**a**) An *N*-port hybrid cloning machine (HCM), consisting of two control knobs: a probabilistic noiseless linear amplifier (NLA) gain (*g*_NLA_) and a deterministic linear amplifier (DLA) gain (*g*_DLA_). Heralded successful events (symbolised by a green light) produce *N* clones (*ρ*_*i*_) of coherent state 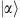 with noise less than the deterministic approach, while unsuccessful events (red light) will be discarded. (**b**) Experimental schematic for HCM. When *g*_NLA_<*g*_DLA_, the cloning machine can be realised by a feed-forward scheme. The input coherent state passes through a beam splitter with transmitivity *T*_s_, where both conjugate quadratures of the reflected port are measured via a dual-homodyne detection setup. The measurement outcomes pass through a heralding function and the successful events are then amplified with gain *g*_x,p_ to displace the corresponding transmitted input state via a strong auxiliary beam. An *N*-port beam splitter finally creates *N* clones, which are characterized by homodyne measurements on quadratures *θ*={*X*, *P*}. 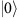, vacuum state; LO, local oscillator; 98:2, 98% transmissive, 2% reflective beam splitter; AM, amplitude modulator; PM, phase modulator.

Our heralded hybrid cloning machine (HCM) is depicted conceptually in Fig. 1a, where an *N*-copy cloner is parametrized by an NLA amplitude gain (*g*_NLA_) and an optimal DLA gain (*g*_DLA_). By introducing an arbitrary input coherent state of |*α*〉, and setting the total gain to unity,


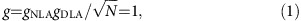


HCM will generate *N* clones with identical complex amplitude *α* and quadrature variance 1+2(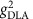
−1)/*N* (where the quantum noise level is 1). Since the probabilistic amplification incurs no noise at all, the variance is a function of *g*_DLA_ only. Such set-up can be interpreted as two linear amplifiers with distinct features, complementing each other by sharing the burden of amplification. Lower noise can be achieved at the expense of the probability of success by increasing the NLA gain. Conversely, a higher probability of success, although with an increased noise, can be obtained by increasing the DLA gain. Hence, by tailoring both gains appropriately, one can achieve the desired cloning fidelity, with vanishing probability of success as the fidelity approaches unity.

**Figure 2:**
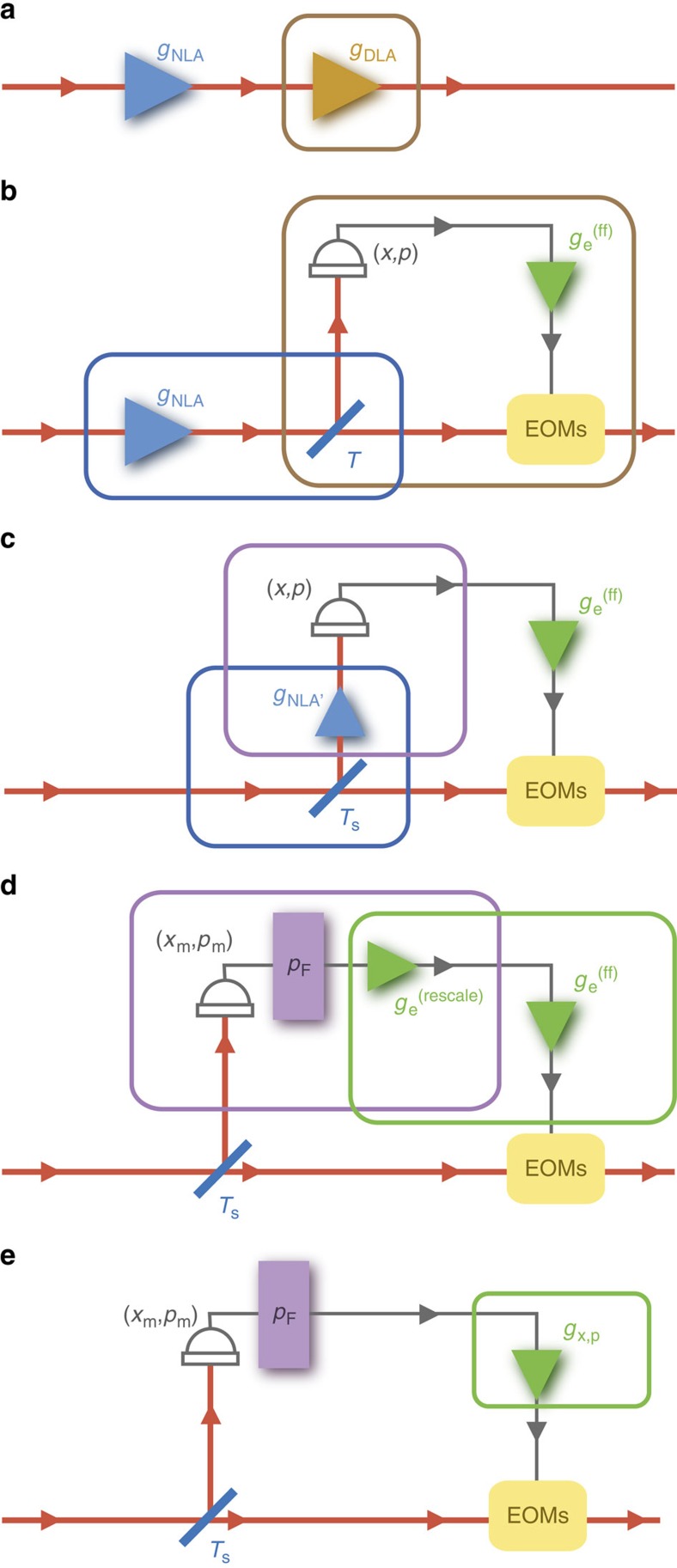
Hybrid Linear Amplifier. (**a**) The general concatenated amplifier, consisting a noiseless linear amplifier (NLA) followed by a deterministic linear amplifier (DLA). (**b**) An optical implementation of the DLA with a beam splitter of transmission *T* and electronic gain 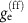. (**c**) When *g*_NLA_<*g*_DLA_, the NLA and beam splitter *T* can be substituted by an effective NLA (NLA′) at the reflection port of a beam splitter *T*_s_. (**d**) The NLA′ followed by a dual-homodyne detection with outcomes (*x*, *p*) is replaced by a heralding function *p*_F_ with an electronic rescaling *g*_e_^(rescale)^ acting upon measurement outcomes (*x*_m_, *p*_m_). (**e**) The two electronic gains are combined into *g*_x,p_. EOMs, electro-optical modulators.

A key feature in our implementation is the observation that when the probabilistic gain is less than the deterministic gain, *g*_NLA_<*g*_DLA_, the hybrid amplifier can be translated to a linear optical setup^35^ with an embedded measurement-based NLA (MBNLA) (Fig. 1b). This equivalence is illustrated in Fig. 2, and discussed in more detail in Supplementary Note 1. The MBNLA is the post-selective version of the physical realization of NLA that has been proposed^37^,^38^, and experimentally demonstrated recently^39^. Compared with its physical counterpart, MBNLA offers the ease of implementation and avoids the predicament of demanding experimental resources. By deploying MBNLA as the heralding function in a feed-forward control set-up^40^, HCM preserves the amplified quantum state, extending the use of the MBNLA beyond point-to-point protocols such as quantum key distribution.

To clone an input coherent state, we first tap off part of the light with a beam splitter of transmission


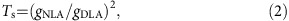


which is then detected on a dual-homodyne detector setup locked to measure the amplitude and phase quadratures (*X* and *P*). A probabilistic heralding function, which is the probabilistic quantum filter function of an MBNLA^37^,^39^, is then applied to the measurement outcome. By post-selecting the dual-homodyne data with higher amplitude, the heralding function gives rise to an output distribution with higher overall mean. Mathematically, this function is given by


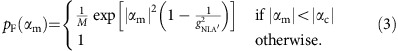


Here, *α*_m_=(*x*_m_+*ip*_m_)/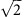 is the dual-homodyne outcomes. *M*=exp[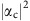
(1−1/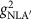)] is the normalization term with *α*_c_ as the NLA cut-off. The NLA gain 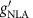 is tailored to achieve unity gain, while the cut-off |*α*_c_| is chosen with respect to the gain and maximum input amplitude to emulate noiseless amplification faithfully while retaining a sufficient amount of data points (Supplementary Note 2).

The heralded signal is then scaled with gain *g*_x,p_=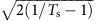 and used to modulate an auxiliary beam. The auxiliary beam is combined with the transmitted beam using a 98:2 highly transmissive beam splitter, which acts as a displacement operator to the transmitted beam^41^. Finally, the combined beam passes through an *N*-port beam splitter to create *N*-clones, which is then verified by homodyne measurements.

**Figure 3:**
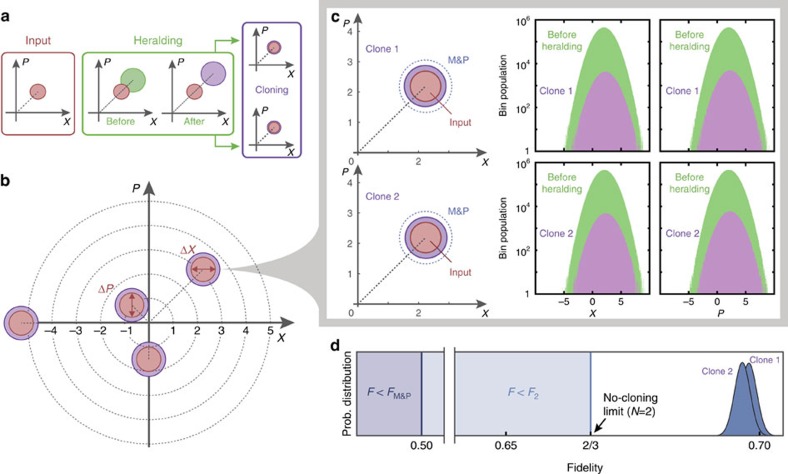
Two clones with hybrid cloning machine. (**a**) Phase space representation of the deterministic-probabilistic hybrid cloning approach. The input state is first deterministically amplified before being heralded to produce a target state which is split into two clones. (**b**) Cloning of distinct input states. Since both the deterministic and noiseless linear amplifiers are invariant to the input state, any unknown coherent state can be cloned in the same way. For a coherent state, the quadrature s.d. Δ*X*=Δ*P*=1. (**c**) Cloning of coherent state (*x*, *p*)=(2.23, 2.19). Left, noise contours (1 s.d. width) of the Wigner functions of the input state (red circle) and the clones from a measure-and-prepare (M&P) cloning machine (dashed blue) and an hybrid cloning machine (purple circle). Right, Quadrature measurement histograms constructed from 5 × 10^7^ homodyne measurements before (green) and 5.9 × 10^5^ measurements after heralding (purple). (**d**) Probability distributions of the fidelity of the clones. Both clones surpass the fidelity limits imposed by the M&P cloner (*F*_M&P_=0.5) and the deterministic cloner (*F*_2_=2/3).

In our experiment, the dual-homodyne measurement heralds successful operation shot-by-shot. This is then paired up with the corresponding verifying homodyne measurements to select the successful amplification events. The accumulated accepted data points give the distribution of the conjugate quadratures of the successful clones. The processing of the input coherent state at different stages of the HCM is illustrated by Fig. 3a.

It is instructive to compare our scheme to that of ref. 36, where probabilistic cloning of fixed-amplitude coherent states was demonstrated. In ref. 36, the amplification is performed by a phase-randomized displacement and phase insensitive photon counting measurement, which have to be optimized according to input amplitude. In our scheme, the amplitude and the phase of the input state is *a priori* unknown. Moreover, owing to the phase-sensitive dual-homodyne measurement and coherent feed-forward control in DLA^27^,^42^, the state to be cloned is amplified coherently in the desired quadrature. The integration of MBNLA in HCM, which emulates a phase-preserving noiseless amplification, further enhances the amplitude of the signal, while maintaining its phase.

### Two clones

To benchmark the performance of the HCM, Fig. 3b demonstrates the universality of the cloning machine by showing the cloning results of four coherent input states with different complex amplitudes 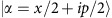, where (*x*, *p*)=(−0.71, 0.72), (−0.01, −1.51), (2.23, 2.19) and (−5.26, −0.02). The figure of merit we use is the fidelity *F*=〈*α*|*ρ*_*i*_|*α*〉, which quantifies the overlap between the input state 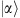 and the *i*-th clone *ρ*_*i*_. Using a setting of *T*_s_≈0.6, our device clones the four input states with average fidelities of 0.695±0.001, 0.676±0.005, 0.697±0.001 and 0.681±0.007, respectively. All of the experimental fidelities are significantly higher than that of a classical measure-and-prepare (M&P) cloner (*F*_M&P_=0.5), where the clones are prepared from a dual-homodyne measurement of an input state^43^. More importantly, all the clones also surpass the no-cloning limit of *F*_2_=2/3, which is impossible even with a perfect deterministic cloning machine.

To further analyse the HCM, we examine the cloning of an input state (*x*, *p*)=(2.23, 2.19) (|*α*|=1.56) in greater detail. This experiment is repeated 5 × 10^7^ times, from which about 5.9 × 10^5^ runs produced successfully heralded clones. The electronic gain *g*_x,p_ and the splitting ratio of the beam splitter are carefully tuned to ensure that the two clones produced are nearly identical. The probabilistic heralding function was chosen to ensure that the output clones have exactly unity gain on average. This is done to prevent any overestimation of the fidelity (Supplementary Note 3 and Supplementary Fig. 1). As can be seen in Fig. 3c, the produced clones have noise significantly lower than the M&P cloning protocol. Since the noise variance is only affected by the deterministic amplification, setting *g*_NLA_>1 will reduce the required DLA gain, while still achieving unity gain. As a result, the clones produced by our HCM will have less noise compared with its deterministic counterpart. The data points also show that the heralded events (purple region of Fig. 3c right) have Gaussian distributions with mean equal to that of the input state. We experimentally obtained a fidelity of 0.698±0.002 and 0.697±0.002 for the two clones. The fidelity plot in Fig. 3d clearly demonstrates that the fidelities of both clones exceed not only the M&P limit but also lie beyond the no-cloning limit by more than 15 s.d.

### Multiple clones

**Figure 4:**
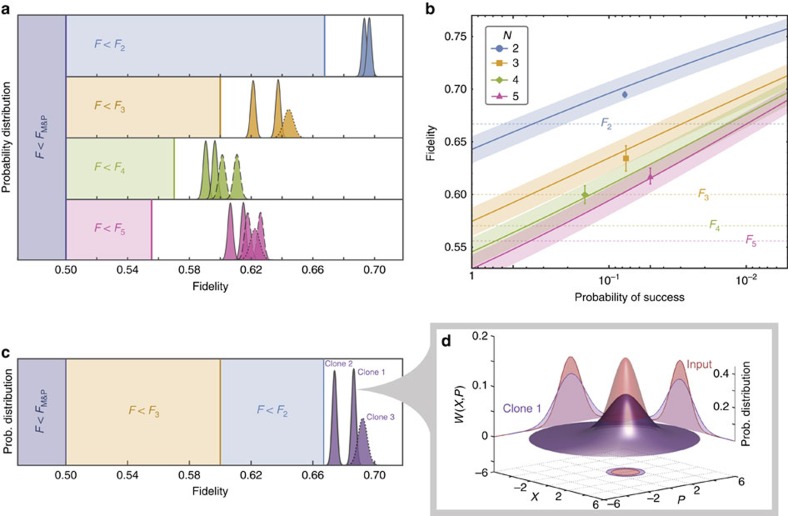
Multiple clones with hybrid cloning machine. (**a**) Fidelity of *N* clones beyond the no-cloning limit. By applying appropriate deterministic and probabilistic gains on the input |*α*|≈0.5, clones with fidelity exceeding their corresponding no-cloning limits *F*_*N*_=*N*/(2*N*−1) are produced. For *N*>2, only two of the output clones are directly measured (solid lines). The remaining *N*−2 clones' fidelity distributions are obtained either from rescaled data of different runs (dashed) or estimation of the remaining intensities (dotted). A sample size of 5 × 10^7^ data points is used for all *N*. The spreads in fidelity distributions are predominately due to imperfect splitting. (**b**) Fidelity as a function of heralding probability of success for different *N*. Theoretical simulations (solid lines) are superimposed with the experimental points (symbols) and the no-cloning limits *F*_*N*_ (dotted lines). Error bars represent 1 s.d. of clones' fidelities and the shaded regions are theoretical expected fidelities from 1 s.d. of the dual-homodyne detection efficiency. (**c**) Three clones with fidelity *F*>*F*_2_ and *F*_3_. (**d**) The experimentally reconstructed Wigner functions of the input (red) and clone 1 (purple) together with their normalized probability distributions for both *X* and *P* quadratures. The quadrature values are normalized to the vacuum.

We operate our HCM at higher gains to enable the production of more than two clones. To have *g*_NLA_>1, from equations (1) and (2), we require *g*_DLA_<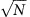, which leads to *T*_s_>1/*N*. Hence, by tailoring *T*_s_ for each *N*, HCM can produce *N* clones with fidelity beating the deterministic bound *F*_*N*_ with the desired probability of success. Figure 4a shows the fidelity of the multiple clones with an input of |*α*|≈0.5. The average fidelities of the clones for *N*=2, 3, 4 and 5 are 0.695±0.002, 0.634±0.012, 0.600±0.009 and 0.618±0.007, respectively, clearly surpassing the corresponding no-cloning limit. In Fig. 4b, we plot the theoretical prediction of the fidelity as a function of the probability of success with the experimental data. The theoretical fidelity is modelled on the dual-homodyne detection efficiency of 90±5%, which is the main source of imperfection (see Supplementary Note 3 for details). We find that our results lie well within the expected fidelities, with the probability of success ranging between 5 and 15%. Remarkably, by keeping 5% of the data points, the average cloning fidelity for *N*=5 can be enhanced by >15%, and hence exceeding the no-cloning limit *F*_5_ by 11.2%.

For deterministic unity gain cloners, as long as *N* clones are produced each with fidelity *F*>*F*_*N*+1_ (refs 12, 13), one may conclude that there are no other clones with equal or higher fidelity. Here we show that this is not necessarily the case for probabilistic cloning. By further increasing NLA gain, we successfully produce three clones, each with fidelity *F*>*F*_2_ (Fig. 4c), and the average fidelity is 0.684±0.009. Given only fidelity, it is impossible for a receiver with only two clones to determine whether the clones originate from a two-clone or three-clone probabilistic protocol (Fig. 4a,c). The resulting probability distribution from 7.2 × 10^6^ successful three-clone states out of 5 × 10^8^ inputs, and the corresponding experimental reconstructed Wigner function are shown in Fig. 4d together with the input state.

The theoretical fidelity for the HCM's clones at unity gain can be shown to be *F*_HCM_=1/(1+(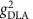
−1)/*N*), which is only a function of the deterministic gain and the number of clones. We note that maximum fidelity for a given *N* can be achieved in the limit of *T*_s_→1, giving *F*_max_(*N*)=1/(1+(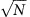
−1)/*N*) (Supplementary Note 3). *F*_max_(*N*) converges to 1 in the limit of an infinite number of clones. However, since this also requires an infinitely large nondeterministic gain, and thus an unbounded truncation in post-selection, the probability of success will be essentially zero.

## Discussion

In summary, we have proposed and demonstrated a hybrid cloning machine that combines a deterministic and a probabilistic amplifier to clone unknown coherent states with fidelity beyond the no-cloning limit. Although an ideal NLA implementation is not possible with our set-up, as this would require zero deterministic gain, our hybrid approach does allow the integration of measurement-based NLA in the optimal deterministic amplifier. We showed that our device is capable of high-fidelity cloning of large coherent states and generation of multiple clones beyond the no-cloning limit, limited only by the amount of data collected and the desired probability of success. Our cloner, while only working probabilistically, provides a clear heralding signal for all successful cloning events.

Several comments on the prospects and avenues for future work are in order. An immediate extension is the implementation of HCM in various feed-forward based cloning protocols, such as phase conjugate cloning^44^, cloning of Gaussian states^45^,^46^, telecloning^47^ and cloning with prior information^36^,^48^,^49^. Our tunable probabilistic cloner could further elucidate fundamental concepts of quantum mechanics and quantum measurement, for instance, quantum deleting^50^ and quantum state identification^34^,^51^. This probabilistic coherent protocol might also play a role in the security analysis of eavesdropping attacks in continuous variable quantum cryptography as well^52^,^53^. The implication of HCM in the context of quantum information distributor^54^ and quantum computation^55^ also demands further investigation.

Beyond probabilistic cloning, owing to the robustness and ease of implementation of this heralded hybrid amplification, we envisage numerous applications in quantum communication^39^,^56^,^57^, quantum teleportation^40^,^58^,^59^ and quantum error correction^60^. As such, we believe our scheme will be a useful tool in the quest to realize large-scale quantum networks.

## Methods

### Experimental details

Our hybrid cloning machine is shown in Fig. 1b. The coherent state is created by modulating the sidebands of a 1,064 nm laser at 4 MHz with a pair of phase and amplitude modulators. In the cloning stage, the input mode is split by a variable beam splitter consisting of a half-wave plate and polarizing beam splitter with transmissivity *T*_s_=(*g*_NLA_/*g*_DLA_)^2^. An optical dual-homodyne measurement is performed on the reflected beam, where the measurement outcome is further split into two parts. The first part is used to extract the 4 MHz modulation by mixing it with an electronic local oscillator, before being low pass filtered at 100 kHz and oversampled on a 12-bit analogue-to-digital converter at 625 k samples per second. The data are used to provide the heralding signal. The second part of the output is amplified electronically with a gain *g*_x,p_ and sent to another pair of phase and amplitude modulators, modulating a bright auxiliary beam. This beam is used to provide the displacement operation by interfering it in phase with the delayed transmitted beam on a 98:2 beam splitter. The delay on the transmitted beam ensures that it is synchronised to the auxiliary beam at the beam splitter. The combined beam is then split by an *N*-port splitter to generate clones. The clones are then verified individually by the same homodyne detector. Two conjugate quadratures *X* and *P* are recorded and used to characterize the Gaussian output. For each separate homodyne detection at least 5 × 10^7^ data points are saved. We note that in evaluating the fidelities, we take into account the detection efficiency and losses to avoid an overestimation of the fidelity (Supplementary Note 3).

### Data availability

The data that support the findings of this study are available from the corresponding author on request.

## Additional information

**How to cite this article:** Haw, J. Y. *et al*. Surpassing the no-cloning limit with a heralded hybrid linear amplifier for coherent states. *Nat. Commun.* **7,** 13222 doi: 10.1038/ncomms13222 (2016).

## Supplementary Material

Supplementary Information Supplementary Figure 1, Supplementary Notes 1-3 and Supplementary References.
