# Supplementary material for: Surpassing the no-cloning limit with a heralded hybrid linear amplifier for coherent states
Source: Nat Commun. 2016 Oct 26;7:13222. doi: 10.1038/ncomms13222 (PMC5095179; doi:10.1038/ncomms13222)
Supplement: Supplementary Information — Supplementary Figure 1, Supplementary Notes 1-3 and Supplementary References. [file ncomms13222-s1.pdf]

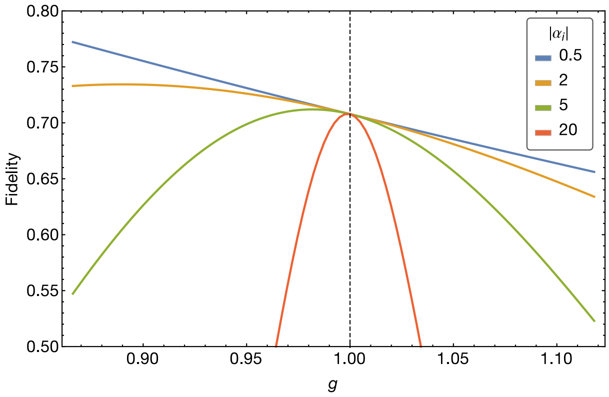

Supplementary Figure 1: **Fidelity of two-clone protocol with different input states.** Deviation from unity gain (dashed line) can lead to overestimation of fidelity when the amplitude of the input state $|\alpha_\mathrm{i}|$ is small.

Supplementary Note 1: Equivalent Theory for Hybrid Cloning Machine

Here we show how concatenating a noiseless linear amplifier (NLA) and deterministic linear amplifier (DLA) (Fig. 2a) can form the amplification stage of an amplify-and-split hybrid cloning machine (HCM). In our scheme, the deterministic amplification can be implemented by a linear optical feed-forwarding circuit [1], which is shown in Fig. 2b. A beam splitter with transmission

$$T = \frac{1}{g_{\mathrm{DLA}}^2}, \tag{1}$$

is used to tap off the noiselessly amplified state. The reflected beam is subjected to a dual homodyne measurement whose outcome $d = (x, p)$ is electronically amplified with gain

$$g_{\mathrm{e}}^{(\mathrm{ff})} = \sqrt{2\left(g_{\mathrm{DLA}}^2 - 1\right)}. \tag{2}$$

This amplified signal is feed-forwarded to the transmitted beam to displace it by $g_{\mathrm{e}}^{(\mathrm{ff})} d$ via electro-optical modulators (EOMs). As described in [2], this same output state can be obtained by a different setup (Fig. 2c) where the NLA is moved from the input to the reflected port with a modified gain

$$g_{\mathrm{NLA}'} = \sqrt{\frac{1-T}{1-T g_{\mathrm{NLA}}^2}} g_{\mathrm{NLA}}, \tag{3}$$

and the beam splitter is replaced by a beam splitter with transmission

$$T_{\mathrm{s}} = T g_{\mathrm{NLA}}^2 = g_{\mathrm{NLA}}^2 / g_{\mathrm{DLA}}^2. \tag{4}$$

These setup are equivalent provided $g_{\mathrm{NLA}} < g_{\mathrm{DLA}}$. The reason for going to this alternate setup is that now we have a situation where the NLA is followed by a dual-homodyne detection which can be implemented by a measurement-based NLA (MBNLA) [3, 4]. The MBNLA consists of a Gaussian heralding function p_{F}, followed by a rescaling factor

$$g_{\mathrm{e}}^{(\mathrm{rescale})} = \frac{1}{g_{\mathrm{NLA}'}}. \tag{5}$$

This rescaling factor is combined with $g_{\mathrm{e}}^{(\mathrm{ff})}$ to give a net electronic gain of

$$\begin{aligned} g_{\mathrm{x,p}} &= g_{\mathrm{e}}^{(\mathrm{rescale})} g_{\mathrm{e}}^{(\mathrm{ff})} \\ &= \sqrt{2\left(\frac{1}{T_{\mathrm{s}}} - 1\right)}, \end{aligned} \tag{6}$$

as shown in Fig. 2e. Finally, subjecting the output to an N-port beam splitter results in a total gain

$$g = g_{\mathrm{NLA}} g_{\mathrm{DLA}} / \sqrt{N} \tag{7}$$

Cloning is achieved when the gain is set to unity, i.e. $g = 1$. Upon setting T_{s} and the number of clones N, the corresponding g_{NLA} and g_{DLA} at unity gain can be determined from Supplementary equation (4). Combining Supplementary equations (4) and (7), we note that as long as $T_{\mathrm{s}} > 1/N$, g_{NLA} will always be bigger than 1, enabling the hybrid operation of the cloning machine.

Supplementary Note 2: Implementation of Measurement-Based Noiseless Linear Amplifier

Here we give a brief summary of the MBNLA implementation in our protocol. The NLA with gain $g_{\mathrm{NLA}'}$ on the reflected mode followed by a dual-homodyne measurement can be replaced by a direct dual-homodyne measurement on the reflected mode, whose outcomes $\alpha_{\mathrm{m}} = (x_{\mathrm{m}} + i p_{m})/\sqrt{2}$ are used to herald successfully cloned states. These measurement data points α_{m} are accepted with probability

$$p_{\mathrm{F}}\left(\alpha_{\mathrm{m}}\right) = \begin{cases} \frac{1}{M} \exp\left[\left|\alpha_{\mathrm{m}}\right|^2 \left(1 - \frac{1}{g_{\mathrm{NLA}'}^2}\right)\right] & \text{if } \left|\alpha_{\mathrm{m}}\right| < \alpha_{\mathrm{c}} \\ 1 & \text{otherwise.} \end{cases} \tag{8}$$

Here α_c is the NLA cut-off and $M = \exp\left[\alpha_c^2\left(1 - 1/g_{NLA'}^2\right)\right]$ ensures that the output is normalized properly. This heralding function, $p_F(\alpha_m)$, together with the rescaling factor (Supplementary equation (5)), can be made arbitrarily close to an ideal NLA operation g^n, where n is the number operator.

Next, we describe how the parameters $g_{NLA'}$ and α_c are chosen in Supplementary equation (8). For both X and P quadratures, the gain $g_{NLA'}$ is chosen such that the inferred mean of N clones prior to splitting equals to that of the amplified input mean to ensure average unity gain. For the quadrature with zero mean, $g_{NLA'}$ will be tuned such that the variance matches the value given by the experimental model with imperfect dual-homodyne detection efficiency. In all cloning protocols, only two clones are directly measured. Based on the N-port splitting ratios, the mean and the variance of the remaining clones for $N > 2$ can be evaluated either from rescaled data from different cloning runs or from an estimation of the remaining transmission power.

The cut-off parameter $\alpha_c > 0$ determines how closely the MBNLA approximates an ideal NLA. A larger cut-off parameter implements the ideal NLA more accurately at the cost of a lower probability of success. The accuracy and the success probability also depend on the $g_{NLA'}$ and the amplitude of the coherent state. The probability distribution of a dual-homodyne measurement on $\rho = |\alpha_0\rangle\langle\alpha_0|$ is given by

$$Q(\alpha_m) = \frac{1}{\pi}\langle\alpha_m|\rho|\alpha_m\rangle = \frac{1}{\pi}\exp(-|\alpha_m - \alpha_0|^2), \tag{9}$$

which is centred around α_0 with the variances $\mathrm{Var}(\mathrm{Re}(\alpha_m)) = \mathrm{Var}(\mathrm{Im}(\alpha_m)) = 0.5$. Applying the probabilistic filter Supplementary equation (8) on the distribution $Q(\alpha_m)$ results in a two-dimensional Gaussian distribution with amplified mean and variance of $g_{NLA'}^2\alpha_0$ and $0.5g_{NLA'}^2$, respectively. To implement the NLA with high fidelity, we propose the following cut-off value for the distribution:

$$\mathrm{Re}(\alpha_c) = g_{NLA'}^2\mathrm{Re}(\alpha_0^{max}) + \beta(\sqrt{0.5}g_{NLA'}) \tag{10}$$

and similarly for $\mathrm{Im}(\alpha_c)$. Here, α_0^{max} is the expected maximum input amplitude involved in the cloning protocols. In our experiment, β is chosen to ensure more than 98% of the data are within the cut-off value for both the two-clone and multi-clone protocols.

Finally, the probability of success for input state $|\alpha_0\rangle$ can be obtained by integrating the function $p_F(\alpha_m)$ (Supplementary equation (8)) with the dual-homodyne distribution $Q(\alpha_m)$.

Supplementary Note 3: Fidelity Evaluation

The fidelity which quantifies the overlap between the clones and the input state is calculated as a criterion for examining the performance of our HCM. We consider single-mode Gaussian input $\rho_i(\mathbf{d}_i, \mathbf{V}_i)$ and output $\rho_o(\mathbf{d}_o, \mathbf{V}_o)$, where $\mathbf{d}_j = (x_j, p_j)$ is the mean of the amplitude and the phase of the state ρ_j while $\mathbf{V}_j = \mathrm{diag}(\sigma_{x_j}, \sigma_{p_j})$ is the corresponding covariance matrix. The fidelity between ρ_i and ρ_o is given by [5]

$$F(\rho_i, \rho_o) = \frac{2}{\sqrt{\triangle + \delta} - \sqrt{\delta}}\exp\left[-\frac{1}{2}\mathbf{d}^T(\mathbf{V}_i + \mathbf{V}_o)^{-\mathbf{1}}\mathbf{d}\right], \tag{11}$$

where $\triangle := \det(\mathbf{V}_i + \mathbf{V_o})$, $\delta := (\det\mathbf{V}_i - 1)(\det\mathbf{V}_o - 1)$, and $\mathbf{d} := \mathbf{d}_o - \mathbf{d}_i$. The fidelity for a coherent state input $\rho_i(\mathbf{d}_i, \mathbf{1})$ is

$$F = \frac{2}{\sqrt{(\sigma_{x_o}^2 + 1)(\sigma_{p_o}^2 + 1)}}\exp\left[-\frac{1}{2}\left(\frac{(x_o - x_i)^2}{\sigma_{x_o}^2 + 1} + \frac{(p_o - p_i)^2}{\sigma_{p_o}^2 + 1}\right)\right].$$

Suppose that the output quadratures are symmetric, and the theoretical output means $\{x_o, p_o\}$ are given by $\{gx_i, gp_i\}$ and the variances $\sigma_{x_o}^2 = \sigma_{p_o}^2 = 1 + 2(g_{DLA}^2 - 1)/N$, respectively. Then, the theoretical fidelity of N clones is thus

$$F(N) = \frac{1}{1 + (g_{DLA}^2 - 1)/N}\exp\left[-\frac{(g-1)^2|\alpha_i|^2}{1 + (g_{DLA}^2 - 1)/N}\right].$$

where $\alpha_i = (x_i + ip_i)/2$. We emphasize that it is crucial to set the gain of the HCM as close to unity gain as possible. As shown in Supplementary Fig. 1, non-unity gain for small input amplitude may lead to an overestimation of the fidelity. At unity gain, the theoretical fidelity reduces to

$$F(N) = \frac{1}{1 + (g_{DLA}^2 - 1)/N}, \tag{12}$$

which depends only on the deterministic gain and number of clones. The maximum fidelity can be achieved in the limit of $T_{\rm s} \to 1$, and from Supplementary equation (4) and (7), $g^2_{\rm DLA} \to \sqrt{N}$, giving

$$F(N) \to F_{\rm max}(N) = \frac{1}{1 + (\sqrt{N} - 1)/N} \; . \tag{13}$$

Note that although the fidelity goes to 1 as N increases, the probability of success is vanishingly small. This is because at this limit, the cut-off $\alpha_{\rm c}$ in the heralding function will also scale with the probabilistic gain $g^2_{\rm NLA}$ to implement the amplification faithfully, thus rejecting essentially most of the data points (c.f. Supplementary equation (8)).

In practice, the fidelity of HCM is limited by several factors, such as the dual-homodyne efficiency, electronic gain, asymmetry in the quadratures and imperfect N-port splitting ratio. Nevertheless, following [6], a simple model of the imperfection of HCM can be constructed simply by considering the detection efficiencies of the dual-homodyne $\eta_{\rm DH}$, which is the dominant source of imperfection. Taking into account the losses and imperfect visibilities, the average dual-homodyne detection efficiency for the amplitude and phase quadratures is $90 \pm 5\%$.

To evaluate the experimental fidelity of the clones, we corrected the homodyne data to ensure proper characterization of both the input state and the clones. This is to avoid overestimation of the fidelity due to underestimation of the clones variances (See Supplementary Fig. 1). The total detection efficiency for the input state is typically around 97%, where we have taken into account the quantum efficiency of the photodiodes, mode-matching visibility, and the propagation losses. The detection efficiency of the clones in the verification stage is about 98.5%.

The correct mean can be obtained by rescaling the overall data by $1/\sqrt{\eta_{\rm tot}}$, where $\eta_{\rm tot}$ is the total detection efficiency of either the input state or the clones. The corresponding variance can be obtained by subtracting $(1 - \eta_{\rm tot})/\eta_{\rm tot}$ from the variance of the rescaled data.

To determine the standard deviation of the fidelity, we take into account the propagation of the uncertainties in the variance of output quadratures. The uncertainty of our fidelity is estimated according to:

$$\mathrm{Var}\left(\sigma^2_{\tilde{F}}\right) = \left(\frac{\partial \tilde{F}}{\partial \sigma^2_{x_{\rm o}}}\right)^2 \mathrm{Var}(\sigma^2_{x_{\rm o}}) + \left(\frac{\partial \tilde{F}}{\partial \sigma^2_{p_{\rm o}}}\right)^2 \mathrm{Var}(\sigma^2_{p_{\rm o}}) \; . \tag{14}$$

Here $\tilde{F}$ is

$$\tilde{F} = \frac{2}{\sqrt{(\sigma^2_{x_{\rm o}} + 1)(\sigma^2_{p_{\rm o}} + 1)}}, \tag{15}$$

which is obtained by setting $x_{\rm o} = x_{\rm i}$ and $p_{\rm o} = p_{\rm i}$ in Supplementary equation (12). The variance of the quadrature variances $\mathrm{Var}(\sigma^2_{x_{\rm o}})$ and $\mathrm{Var}(\sigma^2_{p_{\rm o}})$ are evaluated based on several parameters: number of datapoints, uncertainty of the total detection efficiency and uncertainty of the N-port beam splitter. Finally the standard deviations of the average fidelities are determined from the spread of the fidelity probability distributions.

Supplementary References

[1] Andersen, U. L., Josse, V. & Leuchs, G. Unconditional quantum cloning of coherent states with linear optics. *Phys. Rev. Lett.* **94**, 240503 (2005).

[2] Blandino, R., Barbieri, M., Grangier, P. & Tualle-Brouri, R. Heralded noiseless linear amplification and quantum channels. *Phys. Rev. A* **91**, 062305 (2015).

[3] Fiurášek, J. & Cerf, N. J. Gaussian postselection and virtual noiseless amplification in continuous-variable quantum key distribution. *Phys. Rev. A* **86**, 060302 (2012).

[4] Chrzanowski, H. M. *et al.* Measurement-based noiseless linear amplification for quantum communication. *Nature Photonics* **8**, 333–338 (2014).

[5] Weedbrook, C. *et al.* Gaussian quantum information. *Reviews of Modern Physics* **84**, 621 (2012).

[6] Andersen, U. L. & Leuchs, G. Optical amplification at the quantum limit. *Journal of Modern Optics* **54**, 2351–2356 (2007).
